# Effect of Urolithin A on the Improvement of Circadian Rhythm Dysregulation in Intestinal Barrier Induced by Inflammation

**DOI:** 10.3390/nu16142263

**Published:** 2024-07-13

**Authors:** Yao Du, Xinyue Chen, Susumu Kajiwara, Kanami Orihara

**Affiliations:** School of Life Science and Technology, Tokyo Institute of Technology, Yokohama 226-8501, Japan; du.y.ac@m.titech.ac.jp (Y.D.); chen.x.ac@m.titech.ac.jp (X.C.); kajiwara.s.aa@m.titech.ac.jp (S.K.)

**Keywords:** Urolithin A, IBD, circadian rhythm, clock gene, intestinal barrier, tight junction, IgA

## Abstract

Circadian rhythm plays an important role in intestinal homeostasis and intestinal immune function. Circadian rhythm dysregulation was reported to induce intestinal microbiota dysbiosis, intestinal barrier disruption, and trigger intestinal inflammation. However, the relationship between intestinal microbiota metabolites and the circadian rhythm of the intestinal barrier was still unclear. Urolithin A (UA), a kind of intestinal microbial metabolite, was selected in this study. Results showed UA influenced on the expression rhythm of the clock genes *BMAL1* and *PER2* in intestinal epithelial cells. Furthermore, the study investigated the effects of UA on the expression rhythms of clock genes (*BMAL1* and *PER2*) and tight junctions (*OCLN*, *TJP1*, and *CLND1*), all of which were dysregulated by inflammation. In addition, UA pre-treatment by oral administration to female C57BL/6 mice showed the improvement in the fecal IgA concentrations, tight junction expression (*Clnd1* and *Clnd4*), and clock gene expression (*Bmal1* and *Per2*) in a DSS-induced colitis model induced using DSS treatment. Finally, the Nrf2-SIRT1 signaling pathway was confirmed to be involved in UA’s effect on the circadian rhythm of intestinal epithelial cells by antagonist treatment. This study also showed evidence that UA feeding showed an impact on the central clock, which are circadian rhythms in SCN. Therefore, this study highlighted the potential of UA in treating diseases like IBD with sleeping disorders by improving the dysregulated circadian rhythms in both the intestinal barrier and the SCN.

## 1. Introduction

The circadian rhythm is a natural process that is widely present from bacteria to mammals and regulates most physiological functions in life to repeat roughly every 24 h in response to the day–night cycle [1]. The molecular circadian rhythm is composed of several genes called “clock genes” which produce a 24 h cycle through the transcription–translation feedback loop [2]. The famous clock gene interaction is the Brain and Muscle Arnt-Like 1 (BMAL1)- Circadian Locomotor Output Cycles Kaput (CLOCK) and PERIOD (PER)- CRYPTOCHROME (CRY) transcription–translation feedback loop [3]. In mammals, the circadian rhythm system consists of a central clock which is located in the suprachiasmatic nucleus (SCN) and peripheral clocks which are located in peripheral tissues. The central clock synchronizes the internal circadian rhythm by receiving external light signals and regulates peripheral clocks through neural and hormonal signals, which further modulate physiological behaviors including the sleep–wake cycle, body temperature oscillation, neural activity, and hormonal secretion rhythms [4,5]. In addition, it was reported that external factors can affect the synchronization of the peripheral clock, such as diet, medicine, temperature, and exercise [6].

Circadian rhythm plays an important role in intestinal physiology, including motility, secretion, blood flow, and the integrity of the intestinal barrier [7,8]. The gut immune system also shows the circadian rhythm which regulates different immune responses during active and inactive phases [8]. Moreover, previous studies showed that circadian rhythm disruptions can lead to intestinal barrier damage and immune dysfunction, potentially triggering or exacerbating inflammatory bowel responses [9,10,11]. Additionally, the gut microbiota exhibits circadian fluctuations, which may relate to the host’s food intake and immune regulation. It was reported that circadian rhythm dysregulation could lead to an imbalance in the gut microbiome, further promoting inflammatory bowel disease (IBD) [12]. These studies showed that the circadian rhythm may influence intestinal barrier function by affecting intestinal physiology, immune function, and the intestinal microbiome.

Urolithin A (UA) is a metabolite produced by gut microbiota through the transformation of ellagitannins and ellagic acid (EA), and has demonstrated significant beneficial effects on health. It has been reported that the intake of UA promotes the mitophagy of mitochondria, which intracellular activity declines with aging [13]. Furthermore, long-term consumption of UA has been shown to extend the lifespan of nematodes, increase the endurance of aged mice, and inhibit muscle atrophy [14]. UA has been shown to play a role in reducing intestinal inflammation and in enhancing the function of the intestinal barrier [15]. Additionally, its antioxidant ability is reported to help mitigate some of the damage caused by oxidative stress to the intestinal mucosa [16]. Our recent study showed that UA modulated the clock gene expression rhythm in Mouse Embryonic Fibroblasts (MEFs) and also modified wheel-running activity in mice [17]. However, no study has demonstrated the effects of UA on the circadian rhythm of the intestinal barrier. Thus, this study focused on exploring the effects of UA on the rhythmic expression of intestinal barrier molecules disrupted by IBD inflammation.

## 2. Materials and Methods

### 2.1. Materials

UA for the cell experiments was purchased from Sigma-Aldrich (St. Louis, MI, USA). Dimethyl sulfoxide (DMSO) was purchased from Nacalai Tesque Inc. (Kyoto, Japan). ML385 was purchased from Selleck Chemicals (Houston, TX, USA).

### 2.2. Cell Culture

Caco-2 cells (ATCC# HTB-37TM) were provided by the RIKEN BRC through the National BioResource Project of the MEXT, Japan, and HT-29 cells (ATCC # HTB-38TM) were provided by the European Collection of Authenticated Cell Cultures (ECACC; Salisbury, United Kingdom). Cells were grown in DMEM (WAKO, Tokyo, Japan) supplemented with 10% (*v*/*v*) FBS (HyClone, Ottawa, Canada) and 1% (*v*/*v*) Penicillin–Streptomycin (WAKO, Tokyo, Japan). Cells were incubated at 37 °C and 5% CO_2_. In this study, the cells were passaged once a week, and medium was changed every three days. Before use in experiments, cells were passaged three times following recovery from stock.

### 2.3. Circadian Gene Expression Rhythm Synchronization and Urolithin a Treatment

Caco-2 cells (6.0 × 10^4^ cells/well) and co-culture systems (Caco-2 cells (4.8 × 10^4^ cells/well) and HT-29 cells (1.2 × 10^4^ cells/well)) were cultured in 24-well plates (VIOLAMO (AS ONE), Osaka, Japan) until reaching 80% confluency. The gene expression rhythm of the cells was synchronized using treatment with 100 nM DEX (Nacalai Tesque, Kyoto, Japan) for 2 h. After the 2 h DEX treatment, the culture medium was replaced with fresh DMEM with FBS and PS. After 24 h culture, cells were treated with vehicle (0.1% DMSO) or UA (40, 100 μM) for 30 h. In this study, 24 h after the end of DEX treatment was defined as Circadian Time 0 (CT 0). At CT 0, the Caco-2 cells and co-culture system were treated with TNF-α (5 ng/mL) and IL-17A (1 ng/mL) as the IBD mimic condition. After 30 min of the IBD-conditioned treatment, the medium of the caco-2 cells or co-culture system was changed to DMEM with DMSO, UA, or UA+ML385 to inhibit the Nrf2 pathway.

### 2.4. Total Cell RNA Extraction and Real-Time qPCR

Total RNA extraction was essentially as in Du, Y et al. (2024), with minor modifications [18]. Total RNA was isolated using QIAzol Lysis Reagent (QIAGEN, Hilden, Germany) according to the QIAzol protocol. The total RNA was reverse transcribed using the ReverTra Ace qPCR RT Master Mix with the gDNA Remover kit (Toyobo, Osaka, Japan). Subsequently, 1.2 µL of the transcribed cDNA sample (diluted to 5 ng/μL) was combined with 9.0 µL of the qPCR reaction mixtures. These mixtures contained 300 nM gene-specific primers (Integrated DNA Technologies, Singapore) and Thunderbird SYBR qPCR mix (Toyobo, Japan). Gene expression was analyzed using the Roche LightCycler^®^ 96 System (Roche Diagnostics GmbH, Mannheim, Germany). Gene expression copy numbers were calculated using the standard curve method with *GAPDH* (cell sample) and *18srrna* (mouse sample) as reference genes, and were normalized with an untreated control. All primers used for RT-qPCR analysis are listed in Table 1.

### 2.5. Animals

Seven-week-old female C57BL/6J mice, n = 50, were obtained from Sankyo Labo Service Corporation (Tokyo, Japan). The mice were divided into 2 groups by simple randomization, and were assigned a numerical identifier (e.g., CT0 C1). Mice were bred in specific pathogen-free (SPF) facility at 22 ± 2 °C and 60 ± 5% humidity under a 12 h light (07:00–19:00)–dark cycle (19:00–07:00). Zeitgeber time 0 (ZT0) was designated as lights-on time and ZT12 as lights-off time. AIN-93M was used to feed the mice, being a common diet. Experiments were performed in non-blinded conditions. This study was approved by the Committee for Animal Experimentation at the Tokyo Institute of Technology (approval number: D2022006, approved on 24 June 2022). Animals were treated in accordance with the committee’s guidelines.

### 2.6. Urolithin a Treatment and Dextran Sulfate Sodium Treatment

UA (Jinan Feiteng Pharmaceutical Technology Co., Jinan, China) was diluted in a mixture of 0.5% carboxymethyl cellulose (CMC) and 0.1% TW80 to a suspension of 2 mg/mL, and was stored at 4 °C. The mice were weighed daily, and were orally administered UA emulsion using a flexible oral gavage tube (Natsume Seisakusho, Tokyo, Japan), with a dosing amount of 20 mg/kg/day (n = 25). Oral administration allows for more precise dosing of Urolithin A compared to mixing it into the diet. The vehicle treated group was orally administered an equivalent volume of the mixture of 0.5% CMC and 0.1% TW80 daily (n = 25). After one week of oral administration of UA, the drinking water of the mice was changed to a 4% Dextran Sulfate Sodium (DSS) solution for 3 days to induce inflammation. If the animal died during DSS treatment, this prevented the collection of data.

### 2.7. Feces Collection

Mouse fresh feces were collected to measure fecal IgA concentration. Fresh stool was collected into a 100 μL PBS containing microcentrifuge tubes and incubated on ice. Then, the concentration of stool was changed to 20 mg/mL and homogenized at 13,500× *g* for 10 min. The fecal supernatant was collected and stored at −80 °C for the ELISA assay.

### 2.8. Fecal IgA ELISA Assay

A fecal IgA concentration ELISA assay was performed essentially as in Du, Y et al. (2024) [18]. The concentration was calculated using an IgA Mouse Uncoated ELISA Kit (Thermo Fisher Scientific, Waltham, MA, USA), according to the manufacturer’s protocols.

### 2.9. Tissue Collection

After being euthanized, the mice colon and the SCN parts were collected. Tissues were placed into 1.5 mL microcentrifuge tubes with 400 μL QIAzol Lysis Reagent (QIAGEN, Hilden, Germany) in an ice box. After being homogenized using a micro homogenizer, samples were centrifuged at 12,000× *g* for 15 min at 4 °C. The supernatants were stored at −80 °C for mRNA extraction.

### 2.10. Statistical and Rhythm Analysis

The data in this study are graphically represented as mean values with standard errors and were statistically analyzed with GraphPad Prism (version 9.30; GraphPad Software, Boston, MA, USA). We used the Kolmogorov–Smirnov test or the F-value test or Bartlett’s test to evaluate the normality of data distribution or bias changes. In addition, a Parametric analysis was performed using a one-way ANOVA and Student’s *t*-test. Statistical significance was set at *p* < 0.05, and *p* < 0.2 data are represented.

In order to verify the circadian rhythmicity of gene expression levels, data were also analyzed using nonlinear regression fitting with the cosinor equation using GraphPad Prism [19].

## 3. Results

### 3.1. Urolithin a Affects Clock Gene Expression Rhythms in Intestinal Epithelial Cells

To investigate whether UA influences the clock gene expression rhythms in the intestinal epithelium, Caco-2 cells were used as an intestinal epithelial cell model and clock genes, *BMAL1*, and *PER2* mRNA expression rhythms were examined. Circadian rhythm analysis data of each group are shown in Table 2.

**Figure 1:**
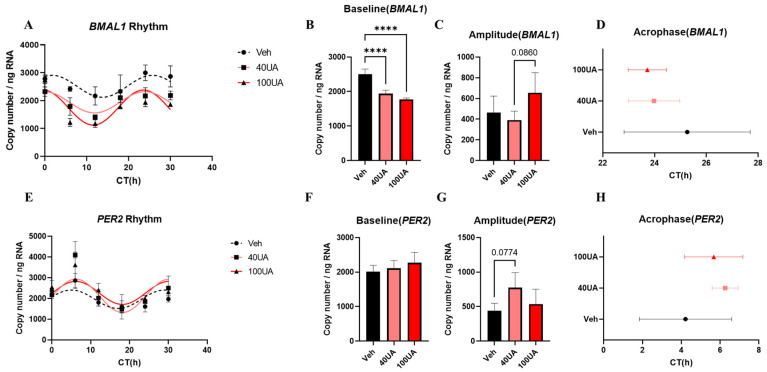
The gene expression of clock genes in Caco-2 cells. The mRNA expression rhythm of *BMAL1* in Caco-2 cells (**A**). The baseline (**B**), amplitude (**C**), and acrophase (**D**) of *BMAL1* mRNA expression rhythm in Caco-2 cells. The mRNA expression rhythm of *PER2* in Caco-2 cells (**E**). The baseline (**F**), amplitude (**G**), and acrophase (**H**) of *PER2* mRNA expression rhythm in Caco-2 cells. Each point represents mean ± SEM, n = 4. Gray represents the vehicle control group while pink (40 μM) and red (100 μM) represent the UA treatment groups. Representative data from two independent trials were shown. **** *p* < 0.0001, unpaired one-way ANOVA between Vehicle, 40UA, 100UA.

qPCR data suggest that *BMAL1* and *PER2* mRNA showed significant circadian oscillation in Caco-2 cells (Figure 1A,E). The UA treatment increased the curve fitting of *BMAL1* and *PER2* expression rhythms based on the R^2^ value (Table 2). The UA treatment reduced the *BMAL1* expression level observed in Caco-2 cells (Figure 1B). The result showed that the amplitude of *BMAL1* tended to be advanced after 100 μM of UA treatment compared with 40 μM of UA treatment (Figure 1C). The *PER2* expression level was not influenced by UA treatment (Figure 1F). However, the amplitude of the *PER2* expression rhythm tended to be advanced after 40 μM of UA treatment (Figure 1G). The acrophase analysis showed that UA treatment may advance the acrophase of *BMAL1* and delay that of *PER2* in Caco-2 cells, but a significant difference was not detected (Figure 1D,H, Table 2).

### 3.2. Urolithin a Improved Circadian Rhythm Dysregulation in Intestinal Epithelial Cells Induced by Inflammation

To investigate the impact of IBD on the circadian rhythms of the intestinal barrier, an in vitro IBD mimic condition was setup by treating Caco-2 cells with pro-inflammatory cytokines TNF-α and IL-17A [20], high levels of which are a key marker of immune dysregulation in IBD patients. The expression level of clock genes and tight junction genes in Caco-2 cells were examined to confirm whether UA treatment has beneficial effects on circadian rhythm dysregulation induced by inflammation in the intestinal barrier. Circadian rhythm analysis data of each group are shown in Table 3 and Table 4.

**Figure 2:**
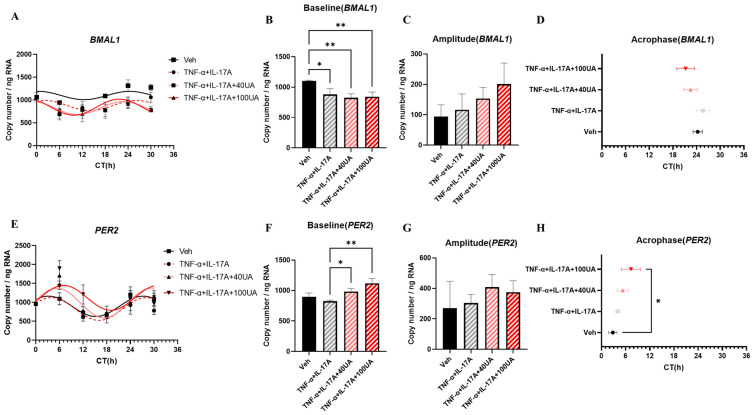
The mRNA expression of clock genes in Caco-2 cells. The mRNA expression rhythm of *BMAL1* (**A**). The baseline (**B**), amplitude (**C**), and acrophase (**D**) of *BMAL1* mRNA expression rhythm in Caco-2 cells. The mRNA expression rhythm of *PER2* (**E**). The baseline (**F**), amplitude (**G**), and acrophase (**H**) of *PER2* mRNA expression rhythm in Caco-2 cells. Each point represents mean ± SEM, n = 3. Representative data from two independent trial were shown. * *p* < 0.05, ** *p* < 0.01, unpaired one-way ANOVA between Vehicle, TNF-α + IL-17A, TNF-α + IL-17A + 40UA, and TNF-α + IL-17A + 100UA.

In our IBD model, *BMAL1* mRNA expression levels showed a decrease after the TNF-α + IL-17A treatment, while *PER2* expression rhythm did not show any change (Figure 2A,E). UA treatment improved the curve fitting of *BMAL1* expression rhythm in TNF-α + IL-17A-treated Caco-2 cells based on the R^2^ value (Figure 2A, Table 3). However, UA treatment did not improve the *BMAL1* expression level in the IBD model (Figure 2B). UA treatment may advance the acrophase of *BMAL1* and increase the amplitude of *BMAL1* compared with the TNF-α + IL-17A-treated group, but did not show a significant difference (Figure 2C,D, Table 2). Additionally, UA treatment improved the curve fitting of the *PER2* expression rhythm based on the R^2^ value, and showed a significant increase in the baseline of the *PER2* expression rhythm (Figure 2E,F, Table 3). A higher dose, 100 μM of UA treatment, also significantly delayed the acrophase of the PER2 expression rhythm (Figure 2H, Table 3). In Caco-2 cells and HT-29 cells’ co-culture system, UA treatment also improved mRNA expression of *CLOCK*, which was enhanced by TNF-α + IL-17A treatment at CT12 (Figure S1A,B). However, although UA treatment showed effects on *CRY1* and *RORA*, these results may not provide conclusive evidence for UA’s role on peripheral clock improvement in the DSS-induced IBD model (Figure S1C–F).

**Figure 3:**
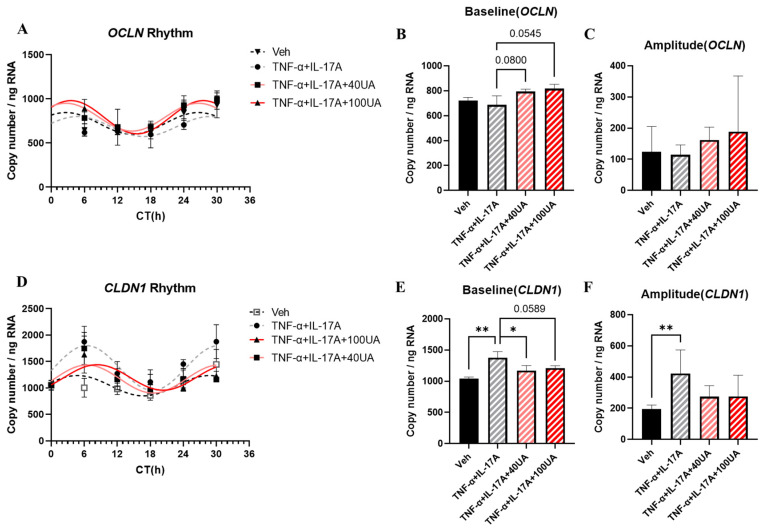
The mRNA expression of tight junction genes in Caco-2 cells. The mRNA expression rhythm of *OCLN* and *CLDN1* (**A**,**D**). The baseline (**B**) and amplitude (**C**) of *OCLN* mRNA expression rhythm in Caco-2 cells. The baseline (**E**) and amplitude (**F**) of *CLDN1* mRNA expression rhythm in Caco-2 cells. Each point represents mean ± SEM, n = 3. Representative data from two independent trials were shown. * *p* < 0.05, ** *p* < 0.01, unpaired one-way ANOVA between Vehicle, TNF-α + IL-17A, TNF-α + IL-17A + 40UA, and TNF-α + IL-17A + 100UA.

Tight junction gene expression results showed that *CLDN1* and *OCLN* expression exhibited 24 h oscillation in Caco-2 cells (Figure 3A,D). TNF-α + IL-17A treatment led to a decrease in the curve fitting of *OCLN* expression rhythm. However, it increased the curve fitting of *CLDN1* based on the R^2^ value (Figure 3A,D, Table 4). The TNF-α + IL-17A treatment also raised the baseline and amplitude of *CLDN1* expression rhythm (Figure 3E). UA treatment demonstrated a significant downregulation of the *CLDN1* expression rhythm curve fitting increase caused by pro-inflammatory cytokines, while significantly lowering the baseline and amplitude of *CLDN1* (Figure 3D–F). Concurrently, UA treatment tended to upregulate the baseline and curve fitting of *OCLN* (Figure 3B, Table 4). UA treatment may also increase the amplitude of *OCLN* compared with the TNF-A + IL-17A group, but did not show significance in its difference (Figure 3C).

### 3.3. UA Pretreatment Improved the Circadian Rhythm in an In Vivo DSS-Induced Colitis Model

In the next experiment, 4% DSS water treatment freely fed for 3 days was used to establish an acute DSS-induced colitis mice model [15]. Mice were treated with 20 mg/kg/day of UA or 0.5% CMC+0.1%TW80 solution as a vehicle treatment at ZT 3 for one week through oral administration before DSS treatment. Mouse feces were collected before and under DSS treatment, and the fecal inflammatory marker IgA was used to determine the effect of UA pre-treatment on IBD. On the final day, colon samples from the mice were collected, and the expression levels of tight junction genes, together with clock genes, were evaluated. The circadian rhythm data of each group are shown in Table 5 and Table 6.

**Figure 4:**
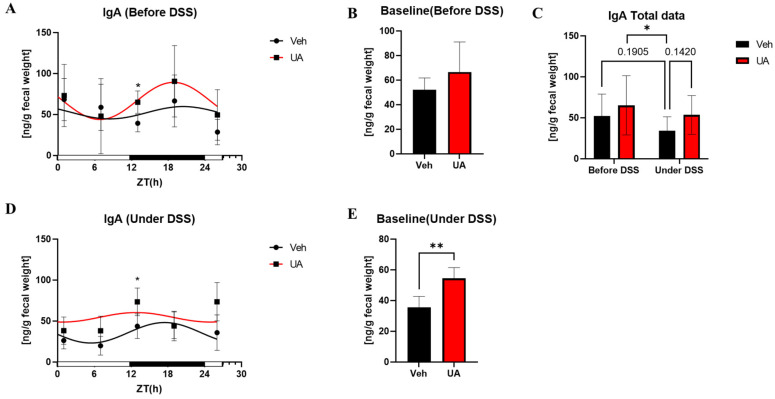
The fecal concentration of IgA before and under DSS treatment. The fecal expression rhythm of IgA before DSS (**A**) and under DSS (**D**). The total fecal IgA concentration (**C**). The baseline (**B**) of IgA concentration rhythm before DSS treatment. The baseline (**E**) of IgA concentration rhythm under DSS. Each point represents mean ± SEM, n = 4, n refers to number of animals. * *p* < 0.05, ** *p* < 0.01 unpaired t-test between Vehicle and UA.

Fecal IgA results exhibited that mice treated with UA for one week showed an increase in fecal IgA concentration and an improvement in the IgA expression rhythm curve fitting based on R^2^ value (Figure 4A, Table 5). UA treatment induced a fecal IgA concentration increase during the dark phase (Figure 4A). Under DSS treatment, the concentration of fecal IgA in the Vehicle-treated group was decreased (Figure 4C). However, the fecal IgA concentration showed an upward trend after UA pretreatment compared with the Vehicle treated group (Figure 4C–E).

**Figure 5:**
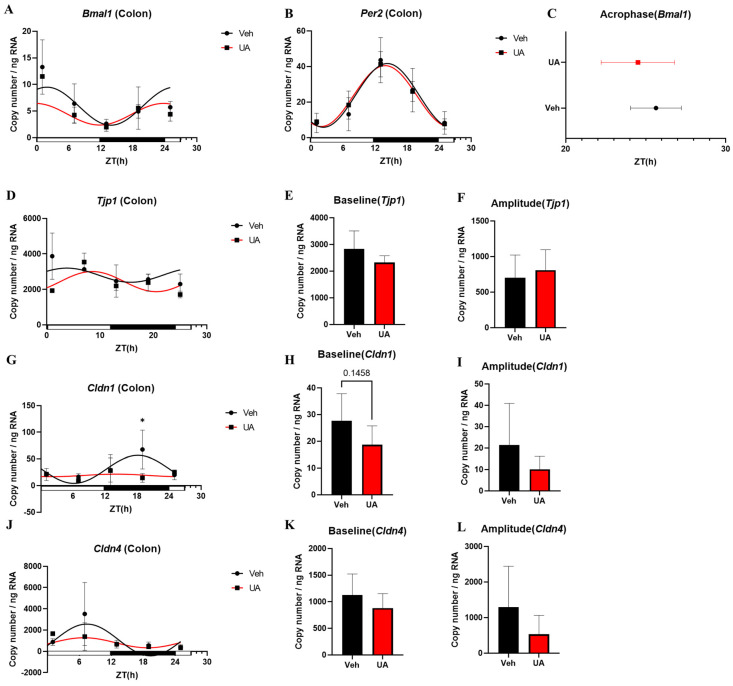
The mRNA expression of clock genes and tight junction genes in mice colon samples. The mRNA expression rhythm of *Bmal1* (**A**) and *Per2* (**B**). The acrophase (**C**) of *Bmal1* mRNA expression rhythm in the colon. The mRNA expression rhythm of *Tjp1* (**D**). The baseline (**E**) and amplitude (**F**) of *Tjp1* mRNA expression rhythm in the colon. The mRNA expression rhythm of *Cldn1* (**G**). The baseline (**H**) and amplitude (**I**) of *Cldn1* mRNA expression rhythm in the colon. The mRNA expression rhythm of *Cldn4* (**J**). The baseline (**K**) and amplitude (**L**) of *Cldn4* mRNA expression rhythm in the colon. Each point represents mean ± SEM, n = 3–5, n refers to number of animals, four mice (DSS group CT2 n = 2, UA group CT0 n = 2) were removed because of death after DSS treatment. * *p* < 0.05, unpaired *t*-test between Vehicle and UA.

Colon sample data show that UA pretreatment did not affect the circadian rhythm of *Bmal1* and *Per2* mRNA expression in the colon (Figure 5A,B). UA treatment may advance the acrophase of *Bmal1* expression rhythm in the colon. However, it did not show a significant difference (Figure 5C, Table 6). UA pretreatment appeared to improve the curve fitting of *Tjp1* expression in the colon after DSS treatment. However, it did not change the baseline and amplitude of the *Tjp1* expression rhythm (Figure 5D–F, Table 6). Interestingly, in this model, UA pretreatment showed a downregulation in the curve fitting of *Cldn1* and *Cldn4* expression (Figure 5G,J, Table 6). UA pretreatment also downregulated the expression level of *Cldn1* in the dark phase and *Cldn4* in the light phase (Figure 5G,J, Table 6).

**Figure 6:**
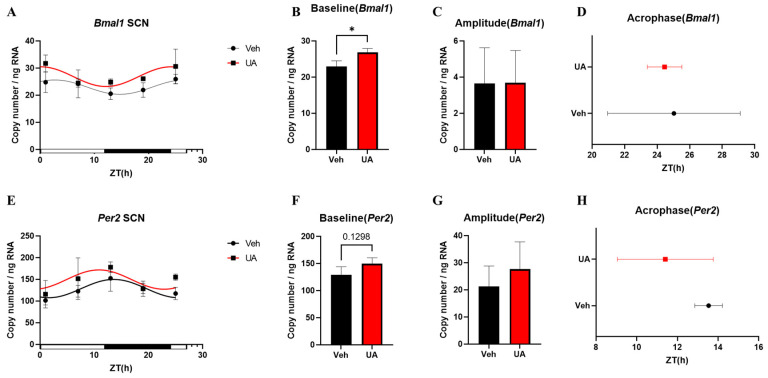
The mRNA expression of *Bmal1* and *Per2* in the SCN. The mRNA expression rhythm of *Bmal1* (**A**). The baseline (**B**), amplitude (**C**), and acrophase (**D**) of *Bmal1* mRNA expression rhythm in the SCN. The mRNA expression rhythm of *Per2* (**E**). The baseline (**F**), amplitude (**G**), and acrophase (**H**) of *Per2* mRNA expression rhythm in the SCN. Each point represents mean ± SEM, n = 3, n refers to number of animals. * *p* < 0.05, unpaired *t*-test between Vehicle and UA.

To determine whether UA pretreatment could affect the central clock, samples from the SCN and surrounding brain tissue were also collected. The circadian rhythm data of each group are shown in Table 7. The results indicate that both *Bmal1* and *Per2* exhibited 24 h expression oscillations, and their circadian rhythms were similar with those observed in the colon samples (Figure 6A,E). After UA pretreatment, the expression levels of *Bmal1* and *Per2* were upregulated (Figure 6B,F). Examination of mRNA expression of other clock-related genes like *Clock*, *Cry1, Per3,* and *Rev-erbα* in mouse SCN also suggested that UA treatment has significantly upregulated clock related gene expression levels (Figure S2A–D). Additionally, UA treatment appeared to advance the acrophase of clock genes, although this effect was not significant (Figure 6D,H, Table 7).

### 3.4. UA Improved the Circadian Rhythm in IBD Model Requires Nrf2 Pathway

According to recent study, the protective effect of UA on the intestinal barrier may be related to the AhR-Nrf2 pathway [17]. ML385 is a novel and specific antagonist of Nrf2 and inhibits the downstream target gene expression of Nrf2. In addition, it was reported that ML385 did not affect growth of lung epithelial cells as high as 25 μM [21]. In this experiment, 25μM ML385 was used to assess the role of the Nrf2 pathway in UA circadian rhythm management. An intestinal epithelial co-culture model was established with Caco-2 cells and HT-29 cells [18] and was stimulated with a combination of cytokines, TNF-α, and IL-17A to mimic the IBD condition. Initially, the rhythmic expression rhythms of the *BMAL1*, *PER2*, and *CLDN1* were examined in this study. Circadian rhythm data of each group are shown in Table 8.

**Figure 7:**
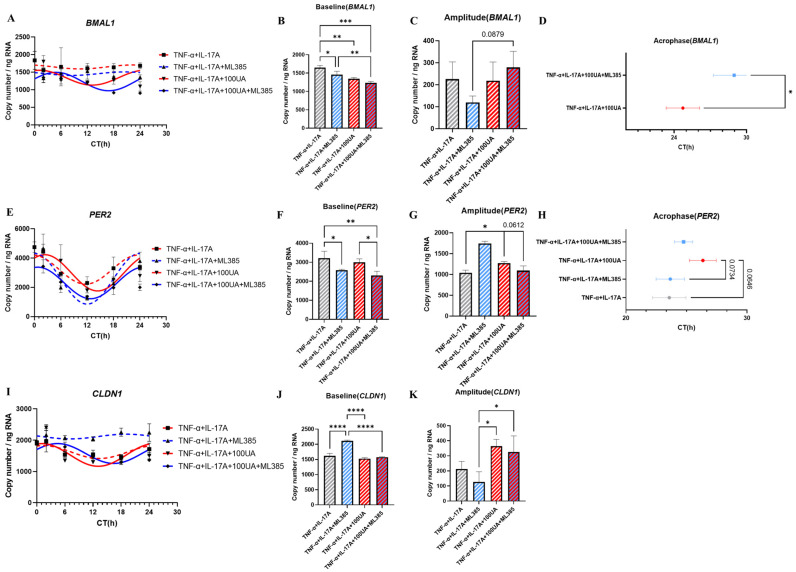
The mRNA expression of *BMAL1*, *PER2*, and *CLDN1* in co-culture system after Nrf2 antagonist treatment. The mRNA expression rhythm of *BMAL1* in the co-culture system (**A**). The baseline (**B**), amplitude (**C**), and acrophase (**D**) of *BMAL1* mRNA expression rhythm in the co-culture system. The mRNA expression rhythm of *PER2* in the co-culture system (**E**). The baseline (**F**), amplitude (**G**), and acrophase (**H**) of *PER2* mRNA expression rhythm in the co-culture system. The mRNA expression rhythm of *CLDN1* in the co-culture system (**I**). The baseline (**J**), amplitude (**K**) of *CLDN1* mRNA expression rhythm in the co-culture system. Each point represents mean ± SEM, n = 3. Representative data from two independent trials were shown. * *p* < 0.05, ** *p* < 0.01, *** *p* < 0.001, **** *p* < 0.0001 unpaired one-way ANOVA between TNF-α + IL-17A, TNF-α + IL-17A + ML385, TNF-α + IL-17A + 100UA, and TNF-α + IL-17A + 100UA + ML385.

The results indicate that the expression rhythm of *BMAL1* was dysregulated after TNF-α + IL-17A treatment and ML385 treatment (Figure 7A). After UA treatment, a recovery in the expression rhythm of *BMAL1* was detected based on R^2^ value (Figure 7A, Table 8). The treatment of UA and ML385 decreased the expression level of *BMAL1* (Figure 7B). In contrast, compared with the UA group, UA and ML385 co-treatment delayed the acrophase of the *BMAL1* expression rhythm (Figure 7D, Table 8). The results for *PER2* exhibited that UA treatment increased the amplitude of the *PER2* expression rhythm and cause a phase delay compared with IBD group (Figure 7G,H). After co-treatment with ML385, the baseline and amplitude of UA-improved *PER2* expression rhythm decreased, and the phase delay induced by UA treatment disappeared (Figure 7E–H, Table 8). Treatment with the ML385 led to an increase in the expression level of *CLDN1* in the co-culture system and disrupted its expression rhythm (Figure 7D–F). In the group treated with both UA and the ML385, the expression level of *CLDN1* decreased, and its expression rhythm was restored (Figure 7D–F, Table 8).

## 4. Discussion

In this study, UA shows the ability to affect intestinal barrier circadian rhythm. UA treatment is found to influence the mRNA expression rhythm of clock genes in Caco-2 cells, including expression level, amplitude, acrophase and curve fitting (Figure 1). Both in in vitro and in vivo models, UA improved the circadian rhythm dysregulation of clock genes and tight junction genes induced by TNF-α + IL-17A treatment and IBD inflammation (Figure 2, Figure 3 and Figure 5). Furthermore, results showed that UA pretreatment enhanced fecal IgA concentration in the dark phase both before and after IBD induction (Figure 4). These results suggest that UA, as one kind of microbial metabolite, can show benefits on the circadian rhythms of intestinal barrier. In addition, different concentrations of UA show varying effects on the amplitude of the rhythmic expression of *BMAL1* and *PER2* (Figure 1B, C,G). These results are consistent with previous research findings in MEFs, demonstrating that the impact of UA on the expression of clock genes is also dose-dependent [17].

By checking the mRNA expression of clock genes in Caco-2 cells under IBD-conditioned treatment, the impact of UA on the circadian rhythms of intestinal epithelial cells in inflammatory conditions are assessed. This study indicates that the circadian rhythmic expression of clock genes in intestinal epithelial cells are disrupted under the influence of pro-inflammatory cytokines (Figure 2). These findings are consistent with the observed decrease in clock gene expression in intestinal mucosa from IBD patients [22]. Additionally, some papers suggest that lifestyle habits that disrupt circadian rhythms in the gut, such as excessive alcohol consumption and social jet lag, contribute to the induction of IBD [23,24,25]. These studies support the finding that the rhythmic expression of clock genes plays a significant role in the development of IBD. In this research, the improvement of *BMAL1* and *PER2* expression rhythm was observed after UA treatment in the IBD model (Figure 2). These data support that UA treatment can improve the disrupted circadian rhythms in intestinal epithelial cells caused by inflammation.

qPCR data also indicate that UA improved the expression of *OCLN* in Caco-2 cells (Figure 3). It is suggested that the expression of tight junctions in the intestinal epithelial barrier has a circadian rhythm and is closely related to clock genes. It is observed that clock gene mutations can downregulate the expression level of TJPs in mice model [26]. In IBD, dysbiosis of tight junctions plays a key role in intestinal barrier disruption. Interestingly, in this study, TNF-α + IL-17A treatment induced the expression of *CLDN1* in Caco-2 cells, while UA treatment inhibited this phenomenon. This result is consistent with reports in IBD, where overexpression of tight junction genes such as *CLDN1*, *CLDN4*, and *CLDN7* is associated with increased intestinal permeability, leading to pathogen invasion [27]. In an in vivo experiment, UA pretreatment also downregulated the expression of *Cldn1* and *Cldn4* after DSS treatment (Figure 5). However, there are few reports on the effects of UA on CLDNs in inflammatory models. A recent report indicates that UA can upregulate TJPs and increase *CLDN1* expression in healthy models [28]. All of these results suggest that more experiments are needed to further clarify UA’s effects on tight junctions in both healthy and inflammatory states. Furthermore, UA is reported to improve tight junction function via the Nrf2-SIRT1 pathway [29]. Thus Nrf2- SIRT1 pathway may play a key role in the amelioration of tight junction expression changes induced by inflammation through UA.

The impact of UA pretreatment on the circadian rhythms of mice intestinal barrier, particularly on the circadian rhythm dysregulation induced by inflammation, were examined. To assess the impact of inflammation, fecal IgA concentration is chosen as inflammatory marker in this study. Results show that UA pretreatment can improve intestinal inflammatory responses by adjusting the expression rhythms of IgA (Figure 4). Inflammatory responses have been confirmed to reduce fecal IgA concentrations [18,30]. Our previous study also indicates that IgA expression exhibits diurnal variations, peaking during active periods [31]. In addition, some studies suggest that the circadian expression rhythm of IgA may be related to gut microbiota composition and host feeding patterns [32,33]. Studies using mouse models with clock gene deletions also indicate that the absence of clock genes can disrupt the circadian rhythm of IgA [32]. These findings suggest that UA pretreatment likely affects the gut microbiota composition and circadian rhythm in mice. Additionally, it is surprising to discover the impact of UA pretreatment on the central clock in the SCN of mice (Figure 6). The SCN can modulate a lot of important physiological functions, such as the sleep–wake cycle. Studies already show that disruptions in sleep patterns can exacerbate the symptoms of IBD, and may even contribute to the development of IBD [9]. In addition, UA was reported to affect *Per2* expression rhythm in mice in the SCN in ex vivo experiment [17]. Currently, no paper shows whether UA can affect the central clock in the SCN in the IBD in vivo model. Although the effect was not very pronounced due to the pre-treatment, these results demonstrate that UA can cross the blood–brain barrier and directly affect the SCN oscillation, which may further improve the sleep disorders in IBD. However, the absence of disrupted expression rhythms of clock genes in colon samples suggests that the intestinal circadian rhythm in this model is still controlled by the central clock. This could be the reason why UA pre-treatment did not affect the expression of clock genes in the colon. These results indicate that the inflammatory model still needs some improvement to show higher effectiveness on the disease control.

In the final experiment, the potential molecular mechanism of UA management of circadian rhythms was confirmed by monitoring the long-term effects of Nrf2 antagonists on a co-culture model of HT-29 and Caco-2 cells. Expression rhythms of *BMAL1*, *PER2*, and *CLDN1* in the HT-29 and Caco-2 co-culture systems are affected after Nrf2 antagonist ML385 treatment (Figure 7). These results suggest that the Nrf2 pathway could be involved in UA’s management of circadian rhythms in intestinal epithelial cells. The Nrf2-SIRT1 pathway is associated with various diseases, including neurodegenerative diseases, cardiovascular diseases, and cancer [34]. SIRT1 also plays a crucial role in the management of circadian rhythm expression. Activation of SIRT1 can promote the binding of E-box to the CLOCK-BMAL1 complex, and facilitate the deacetylation and degradation of PER2 in the cytoplasm [35]. These results suggest that UA may alter the expression rhythms of target genes through the Nrf2-SIRT1 pathway. Additionally, recent study shows that, in NrF2-/- mice, high concentration UA treatment can still inhibit inflammatory responses [36]. A previous report also indicates that nobiletin, a compound with a similar structure to UA, acts as an agonist of the ROR and can enhance the circadian rhythm amplitude of *PER2* [37]. These results suggest that UA treatment may influence the circadian rhythm in the intestinal barrier through multiple pathways.

## 5. Conclusions

In conclusion, the ability of UA to influence the expression of clock genes in intestinal epithelial cells was confirmed in this study. By inducing inflammation in an intestinal epithelial cell model and a mouse model, the current study summarizes UA with activities in mitigating IBDs by improving the circadian expression rhythm of the clock gene and tight junction genes in the intestinal barrier. In addition, UA can improve the intestinal immune system by modulating immune responses rhythms such as IgA expression. The relationship between UA and the Nrf2-SIRT1 pathway may be partly responsible for these results. Overall, the influence of UA on the circadian rhythms of the intestinal barrier holds not only potential for treating IBD, but also may be significant for metabolic diseases caused by modern lifestyle factors such as shift work and social jet lag.

## Figures and Tables

**Table 1:** Primer list.

Gene	Primer Sequence	
	Forward	Reverse
(Human)		
*GAPDH*	CAGCCTCAGTACAGCAATCAAC	TAGGGGTCATAGGAGTCATTGG
*BMAL1*	GTGGACTTGACACCTCTTCT	GGAGCATAGCAGGGAGTTT
*PER2*	CTGCTAATGTCCAGTGAGAG	GTACAGGATCTTCCCAGAAAC
*CLDN1*	GGTGCTATCTGTTCAGTGATG	GGCTGACTTTCCTTGTGTAG
*OCLN*	GCTTCAGTTGGTGTTGTGAG	GATGGCATGGTGTAGTGTAG
(Mice)		
*18srrna*	CGAAAGCATTTGCCAAGAAT	GCGGGTCATGGGAATAAC
*Bmal1*	CACCAACCCATACACAGAAG	GACAGACTCGGAGACAAAGA
*Per2*	GATGTGACAGGCTGTGTTTA	GTTCACCTTCCTCCTCTTTG
*Cldn4*	GATGGCGTCTATGGGACTAC	CGCACAACTCAGGATGATCC
*Tjp1*	CAGAGTTTGACAGTGGAGTT	CCATCCTCATCTTCATCTTCTT
*Cldn1*	GCAATGTTTGTGTCCACCATT	GTCGCCAGACCTGAAATTAAA

**Table 2:** Circadian rhythm data of *BMAL1* and *PER2* mRNA expression in each group.

	Group	Baseline	Amplitude	Acrophase	R^2^
*BMAL1*	Vehicle	2513	393.6	25.26	0.4219
	40 μM UA	1940	381.0	23.97	0.5545
	100 μM UA	1751	627.7	23.72	0.6866
*PER2*	Vehicle	1961	444.6	4.21	0.3205
	40 μM UA	2126	805.0	6.26	0.5222
	100 μM UA	2267	555.8	5.67	0.3670

R^2^ value shows fitting to the cosinor curve, varies from 0 (worst) to 1 (best).

**Table 3:** Circadian rhythm data of clock genes mRNA expression in each group.

	Group	Baseline	Amplitude	Acrophase	R^2^
*BMAL1*	Vehicle	1142.0	93.8	24.29	0.1059
	TNF-α + IL-17A	880.1	116.3	25.62	0.2044
	TNF-α + IL-17A + 40 μM UA	824.7	153.5	22.47	0.4025
	TNF-α + IL-17A + 100 μM UA	843.8	200.9	21.21	0.5621
*PER2*	Vehicle	894.2	271.0	2.83	0.482
	TNF-α + IL-17A	824.0	303.5	4.06	0.563
	TNF-α + IL-17A + 40 μM UA	981.3	408.5	5.29	0.214
	TNF-α + IL-17A + 100 μM UA	1117.0	375.0	7.31	0.609

R^2^ value shows fitting to the cosinor curve, varies from 0 (worst) to 1 (best).

**Table 4:** Circadian rhythm data of tight junction genes mRNA expression in each group.

	Group	Baseline	Amplitude	Acrophase	R^2^
*CLDN1*	Vehicle	1043	268.1	4.72	0.5170
	TNF-α + IL-17A	1378	422.3	6.42	0.6090
	TNF-α + IL-17A + 40 μM UA	1169	265.2	6.51	0.4167
	TNF-α + IL-17A + 100 μM UA	1198	240.5	8.20	0.3989
*OCLN*	Vehicle	722.6	122.8	2.43	0.3802
	TNF-α + IL-17A	686.9	113.4	4.89	0.2690
	TNF-α + IL-17A + 40 μM UA	793.5	157.5	2.52	0.6561
	TNF-α + IL-17A + 100 μM UA	792.5	187.8	3.51	0.4770

R^2^ value shows fitting to the cosinor curve, varies from 0 (worst) to 1 (best).

**Table 5:** Circadian rhythm data of fecal IgA concentration before and after DSS treatment.

	Group	Baseline	Amplitude	Acrophase	R^2^
Before DSS	Vehicle	52.13	-	-	0.0339
	UA	66.60	13.81	18.51	0.1717
Under DSS	Vehicle	35.64	8.75	17.80	0.2609
	UA	54.53	-	-	0.0343

R^2^ value shows fitting to the cosinor curve, varies from 0 (worst) to 1 (best).

**Table 6:** Circadian rhythm data of clock genes and tight junction genes’ mRNA expression in colon.

	Group	Baseline	Amplitude	Acrophase	R^2^
*Bmal1*	Vehicle	5.92	3.79	24.18	0.3792
	UA	4.41	3.54	21.17	0.2479
*Per2*	Vehicle	23.76	19.13	13.93	0.6991
	UA	23.37	17.56	13.88	0.8219
*Tjp1*	Vehicle	2801.0	-	-	0.0917
	UA	2441.0	809.0	9.27	0.3063
*Cldn1*	Vehicle	27.3	21.5	26.80	0.2111
	UA	19.1	-	-	0.0123
*Cldn4*	Vehicle	1074.0	1291.0	8.81	0.3328
	UA	808.0	528.0	8.53	0.1804

R^2^ value shows fitting to the cosinor curve, varies from 0 (worst) to 1 (best).

**Table 7:** Circadian rhythm data of *Bmal1* and *Per2* mRNA expression in the SCN.

	Group	Baseline	Amplitude	Acrophase	R^2^
*Bmal1*	Vehicle	745.9	180.9	24.40	0.6159
	UA	640.4	87.7	26.51	0.4970
*Per2*	Vehicle	790.4	151.7	13.53	0.8299
	UA	1049.0	315.3	11.60	0.8783

R^2^ value shows fitting to the cosinor curve, varies from 0 (worst) to 1 (best).

**Table 8:** Circadian rhythm data of *BMAL1*, *PER2*, and *CLDN1* mRNA expression in each group.

	Group	Baseline	Amplitude	Acrophase	R^2^
*BMAL1*	TNF-α + IL-17A	1650	225.7	-	0.0295
	TNF-α + IL-17A + ML385	1459	119.4	-	0.0193
	TNF-α + IL-17A + 100 μM UA	1344	218.6	24.72	0.2368
	TNF-α + IL-17A + 100 μM UA + ML385	1234	279.1	28.97	0.2411
*PER2*	TNF-α + IL-17A	3215	1038	23.58	0.6285
	TNF-α + IL-17A + ML385	2588	1745	23.66	0.8300
	TNF-α + IL-17A + 100 μM UA	2995	1272	26.35	0.5372
	TNF-α + IL-17A + 100 μM UA + ML385	2304	1093	24.76	0.4674
*CLDN1*	TNF-α + IL-17A	1622	213.3	2.79	0.5042
	TNF-α + IL-17A + ML385	2118	127.4	-	0.0687
	TNF-α + IL-17A + 100 μM UA	1529	364.0	2.85	0.4456
	TNF-α + IL-17A + 100 μM UA + ML385	1575	324.0	1.98	0.4471

R^2^ value shows fitting to the cosinor curve, varies from 0 (worst) to 1 (best).

## Data Availability

The original contributions presented in the study are included in the article/Supplementary Material, further inquiries can be directed to the corresponding author.

## Supplementary Materials

The following supporting information can be downloaded at: https://www.mdpi.com/article/10.3390/nu16142263/s1, Figure S1: The mRNA expression of clock genes in Caco-2 cell and HT-29 cell co-culture system; Figure S2: The mRNA expression of clock related genes in the SCN.
