# Supplementary material for: Effect of Urolithin A on the Improvement of Circadian Rhythm Dysregulation in Intestinal Barrier Induced by Inflammation
Source: Nutrients. 2024 Jul 13;16(14):2263. doi: 10.3390/nu16142263 (PMC11280374; doi:10.3390/nu16142263)
Supplement: Supplementary file 1 [file nutrients-16-02263-s001.zip › nutrients-3031899-supplementary.pdf]

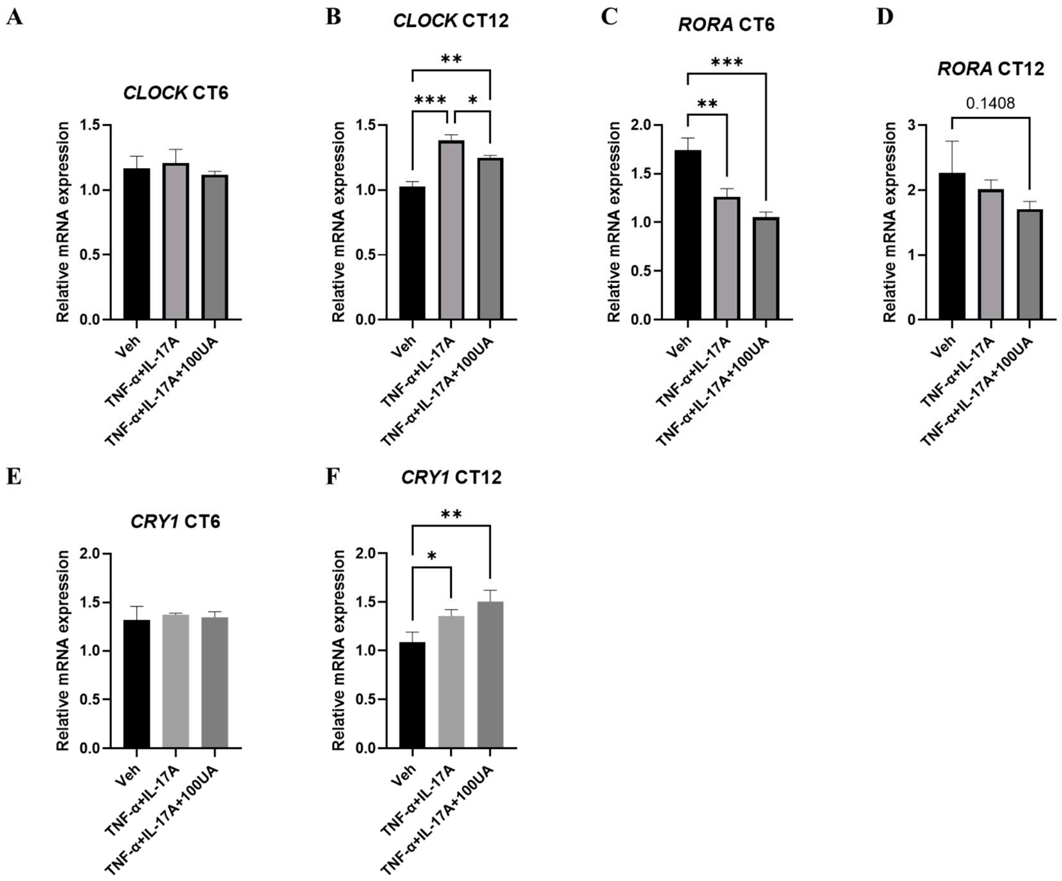

Figure S1. The mRNA expression of clock genes in Caco-2 cell and HT-29 cell co-culture system The mRNA expression rhythm of *CLOCK*(A&B), *RORA*(C&D), and *CRY1*(E&F). Each point represents mean ± SEM, n = 3. *$p < 0.05$, ** $p < 0.01$, *** $p < 0.001$ unpaired one-way ANOVA between Vehicle, TNF-α + IL-17A, TNF-α + IL-17A +100UA.

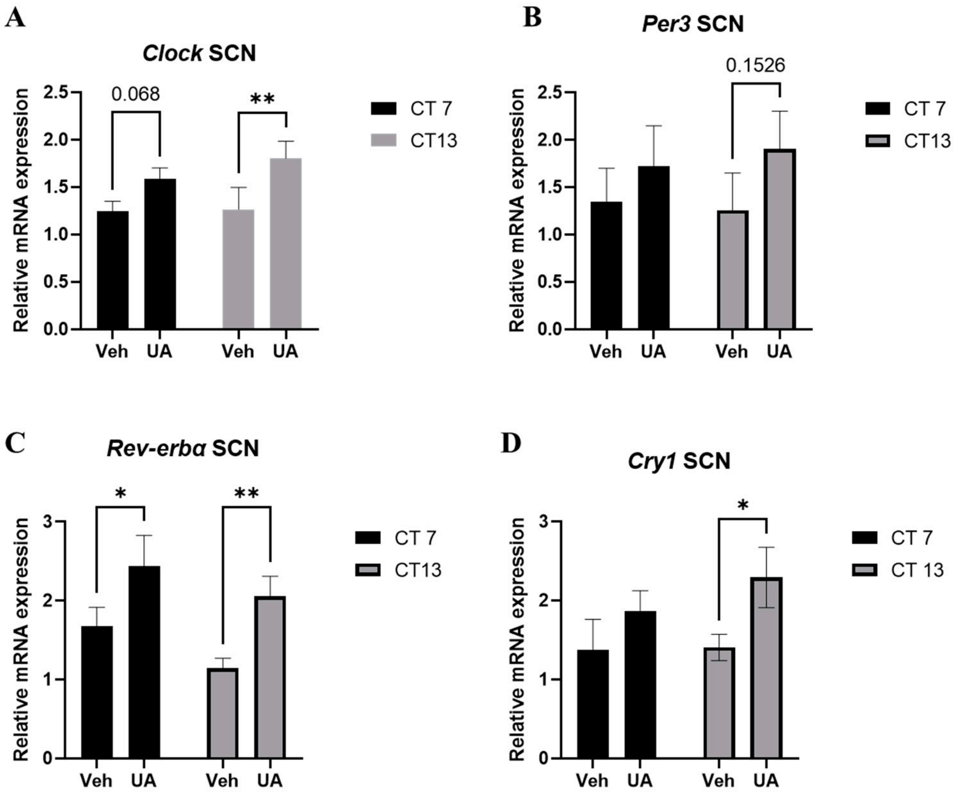

Figure S2. The mRNA expression of clock related genes in the SCN The mRNA expression level of Clock(A), Per3(B), Rev-erbα(C), and Cry1(D). Each point represents mean ± SEM, n = 3, n refers to number of animals. * $p < 0.05$, ** $p < 0.01$, unpaired t-test between Vehicle and UA.
